# The Home Production of Food

**Published:** 1915-11-27

**Authors:** 


### THE HOME PRODUCTION OF FOOD.

The Departmental Committee which was appointed in June last to consider what steps should be taken for the sole purpose of maintaining and, if possible, increasing the present production of food in England and Wales, on the assumption that the war may be prolonged beyond the harvest of 1916 has now issued its final report. The Committee is of opinion that there is great need to increase the productivity of the soil of our country by stimulating more intensive cultivation, and by bringing under the plough a large area of land at present wastefully devoted to inferior pasture. The question naturally arises whether arable land in the occupation of hospital authorities could be utilised for the increased production of food. The answer is undoubtedly in the affirmative, and The Hospital has long advocated the planning and cultivation of hospital gardens on an extensive and systematic scale. And while there is nothing in the present Report which can add anything to the arguments that have been used in these columns in favour of the cultivation of such gardens, the recommendations of the Committee certainly strengthen the position THE HOSPITAL has always taken up in regard to this matter.

When the Committee was appointed there was at least a suspicion that the activity of hostile submarines might endanger our supplies of foodstuffs from overseas and therefore render it essential that every plot of land should be placed under cultivation. But this danger is no longer a real one, and, as Lord Inchcape and Sir Harry Verney point out in a Minority Report, the submarine menace is apparently "well in hand"; the Government are not now afraid of a short supply of wheat from abroad, and in these circumstances it is hardly necessary that any extraordinary measures should be taken to ensure a home-grown supply, even if the war should extend beyond the autumn of 1916. On the other hand, it is the view of the majority of the Committee that the intensification of our agriculture will be even more necessary after the war than now, for then the nation's indebtedness will have reduced its purchasing power abroad, and the need will be felt for the extra employment of labour that arable land provides. If this view is accepted as the correct one, it is clearly in the interests of the country that wherever practicable grass land should be broken up for the purpose of growing wheat, and that unused land in the neighbourhood of villages and the suburbs of towns should be utilised for producing vegetables and other foods. The part which hospital authorities could play in any great national scheme would naturally be a comparatively small one, but if hospitals with land at their disposal would follow the Exeter example described in THE HOSPITAL of September 18 (p. 529) they would at least be in a position to supply their own needs in the way of fresh vegetables and fruits, and so add something to the country's supplies of foodstuffs.

